# Long‐term adherence to glucose‐lowering medications in adults with diabetes: A data linkage study

**DOI:** 10.1111/dom.16408

**Published:** 2025-04-23

**Authors:** Crystal M. Y. Lee, Alice A. Gibson, Natasha Nassar, Stephen Colagiuri

**Affiliations:** ^1^ School of Population Health Curtin University Perth Western Australia Australia; ^2^ Boden Initiative, Charles Perkins Centre and Faculty of Medicine and Health University of Sydney Sydney New South Wales Australia; ^3^ Menzies Centre for Health Policy and Economics, School of Public Health, Faculty of Medicine and Health University of Sydney Sydney New South Wales Australia

**Keywords:** diabetes, dispensing records, medication adherence

## Abstract

**Aims:**

To determine the adherence rate in users of each class of glucose‐lowering medication and identify the key socio‐demographic factors influencing adherence.

**Methods:**

The 45 and Up Study is an ongoing cohort study of residents aged ≥45 years in New South Wales, Australia. We analysed Pharmaceutical Benefits Scheme records from 2013 to 2019 of the 21 341 study participants who self‐reported having diabetes. Medication adherence was estimated as the proportion of days covered for each 12‐month period for up to the fifth 12‐month period.

**Results:**

A consistent pattern was observed across all drug classes, where the percentage of adherent (proportion of days covered ≥0.8) users was highest in the first 12 months, followed by a drop in the second 12 months. For prevalent users on the same drug class for the full 5‐year period, higher percentages of adherent users compared to the first 12 months were observed for glucagon‐like peptide 1 receptor agonists (77.7% vs 74.2%). For incident users on the same drug class for the full 5‐year period, a higher percentage of adherent users compared to the first 12 months was observed for sodium‐glucose cotransporter 2 inhibitors (84.2% vs 78.4%). Moreover, no socio‐demographic subgroup was consistently more or less adherent to medications.

**Conclusions:**

Initial adherence was good and remained relatively high over time in this cohort. Nevertheless, adherence was still a challenge in some individuals. Practitioners should recognize the possibility of non‐adherence and consider this at each consultation.

## 1 INTRODUCTION

Diabetes is a major cause of cardiovascular disease, chronic kidney disease, lower limb amputation, and blindness. Suboptimal blood glucose control significantly contributes to these complications. Research consistently shows that improving blood glucose management can delay the onset of these complications, with glucose‐lowering medications (GLMs) playing a critical role in maintaining optimal blood glucose levels.^1^ However, a major barrier to achieving effective glucose control is the widespread issue of medication non‐adherence, which undermines the potential benefits of these treatments.^2^, ^3^

In Australia, despite approximately three‐quarters of the population with diabetes receiving government‐subsidized GLMs, just over half of the adult population with known diabetes achieves the recommended glycated haemoglobin (HbA1c) target of <53 mmol/mol (7.0%).^4^, ^5^, ^6^ Previous Australian studies have attempted to measure adherence to GLMs using survey data, self‐reported tools, and pharmacy medication dispensing records.^7^, ^8^ However, these studies often suffered from small sample sizes, limiting the generalisability of the findings. Additionally, while several studies have examined the relationship between various factors such as age, depression, healthcare costs and medication adherence, the results have been inconsistent,^9^ potentially due to differences in study settings such as those involving universal healthcare versus user‐pays healthcare systems.

To effectively address and improve medication adherence, it is crucial to identify factors influencing adherence among individuals with diabetes in Australia. This study, therefore, aimed to determine the adherence rate for each class of GLM and identify the key socio‐demographic factors influencing adherence in the middle‐aged and older population with diabetes in Australia. Understanding these factors will be essential for developing targeted interventions to enhance medication adherence and, consequently, improve health outcomes for those living with diabetes.

## 2 METHODS

### 2.1 Participant

The Sax Institute's 45 and Up Study is an ongoing cohort study of residents aged ≥45 years in New South Wales (NSW), Australia.^10^ In 2005–2009, residents were randomly sampled from the Services Australia Medicare enrolment database with oversampling in people aged ≥80 years and those who lived in rural and remote areas. Altogether, 267 357 individuals (11% of the NSW population aged ≥45 years) completed the baseline questionnaire and consented to follow‐up and linkage of their information to health and other databases (response rate 19%). In 2010, the first 100 000 enrolled participants were sent the Social, Economic and Environmental Factors Study questionnaire for completion (response rate 60%). In 2012–2015, all participants were sent a follow‐up questionnaire for completion (response rate 58%).

The NSW Centre for Health Record Linkage used probabilistic matching to link questionnaires of participants with their NSW Registry of Birth, Deaths and Marriages mortality records, with records with an uncertain probability of being true matches checked by hand. Its current estimated false positive rate is 0.5% (http://www.cherel.org.au). Pharmaceutical Benefits Scheme (PBS) claims records supplied by Services Australia were linked using deterministic matching.

We included participants for analysis if they answered ‘Yes’ to the question ‘Has a doctor ever told you that you have diabetes?’ in any of the questionnaires (https://www.saxinstitute.org.au/solutions/45‐and‐up‐study/use‐the‐45‐and‐up‐study/data‐and‐technical‐information/); were alive on 1 January 2013; and had at least one PBS dispensing record of any GLM since 1 January 2013. Participants who withdrew from the study or only dispensed acarbose and/or insulin were excluded from the analysis.

### 2.2 Medication dispensing data

The PBS records the supply of medications subsidized by the Australian government. However, not all subsidized medications were captured until July 2012.^11^ We, therefore, excluded records before 1 July 2012. Subsidized GLMs included insulin (Anatomical Therapeutic Chemical classification code^12^: A10A), metformin (A10BA02), sulfonylureas (A10BB), acarbose (A10BF01), thiazolidinedione (A10BG), dipeptidyl peptidase‐4 inhibitors (DPP‐4i; A10BH), glucagon‐like peptide 1 receptor agonists (GLP‐1RA; A10BJ and A10BX (before 2017)), sodium‐glucose cotransporter 2 inhibitors (SGLT2i; A10BK and A10BX (before 2017)) and their combinations (A10BD).

### 2.3 Definitions of medication use status

PBS records between 1 July and 31 December 2012 were used to determine baseline medication use status by drug class (Figure S1). Participants with at least one dispensing record of a drug class during this period were considered *prevalent users* of that drug class. *Incident users* were participants with at least one dispensing record of the drug class after 31 December 2012 who did not qualify as a prevalent user of that drug class. Participants were considered to have discontinued a drug class if the period between two consecutive records of the same drug class was greater than 12 months and there was no record of another drug class to indicate a switch in drug class. Medication switch was considered to have occurred when the date of supply of another drug class was greater than the last date of supply of the drug class being studied and the participant was alive over the 12‐month period being studied.

### 2.4 Statistical analysis

All medication adherence analyses were conducted on PBS records from 1 January 2013 to 31 December 2019. Only participants with at least two records of a drug class within 12 months were included in the adherence analysis. Nevertheless, we reported the percentage of participants with only one dispensing record of a drug class since discontinuation after one script may indicate drug intolerance. Medication adherence was estimated by calculating the proportion of days covered (PDC), which is the number of days with medication on hand divided by the number of days in the 12‐month period being studied.^13^ Adherence was defined as PDC ≥0.8 (i.e., filling of prescription enough to cover 80% of the time based on the standard coverage days (SCD) for the specific drug class). SCD was defined as the median time to re‐supply by any item of the same drug class. We applied SCD for drug classes presented in the PBS Drug utilization sub‐committee 2017 report.^5^ Based on the date of supply of a drug class first recorded since 1 January 2013 (index date), a PDC was calculated for each 12‐month period for up to the fifth 12‐month period. The end of the study period was either 31 December 2019, date of death or last date of supply plus the SCD for those who discontinued or switched the drug class, whichever came first. Participants who reached the end of the study prior to a full 12‐month period were included for partial 12‐month analysis along with participants who had the full 12‐month dispensing record for that period. Therefore, the mean analysis days for any 12‐month period would be less than 365 days if some participants included in the analysis in that period had dispensed the same drug class for less than the full 12 months. Records of a drug class that appeared after a gap of more than 12 months (i.e., restarted the drug class) were excluded from analysis. For participants who were on the same drug class continuously for at least 5 years, we also calculated the 5‐year PDC and 5‐year per cent adherent.

PDC was not calculated for insulin, as the daily dosage varies within and between users and for acarbose, as the sample size was small. Dispensing records of insulin and acarbose were only used to determine whether a participant discontinued or switched a drug class. Furthermore, unlike other drug classes where one tablet per day is taken, the number of tablets per day taken for metformin and sulfonylurea can differ between participants except for fixed‐dose combinations that include metformin and/or sulfonylurea. An issue with estimating PDC for metformin and sulfonylurea is that their SCD are 37 and 33, respectively. However, for individuals prescribed a dosage of one 500 mg tablet per day, for example, a box containing 100 metformin 500 mg tablets, the supply would last almost three times longer compared to those instructed to take 1500 mg per day (equivalent to three tablets per day). We, therefore, included subgroup analyses for prevalent users by metformin type (i.e. metformin alone or metformin fixed‐dose combination) and alternative SCD of 60 in the metformin alone group to address this issue. Metformin fixed‐dose combinations that were available included metformin‐sulfonylurea, metformin‐DPP‐4i, metformin‐thiazolidinedione and metformin‐SGLT2i.

Multiple logistic regression was used to compare the percentage of adherent users between subgroups for each drug class separately for prevalent and incident users. The models were adjusted for sex, age group in 2013, duration of diabetes in 2013, socio‐economic status at baseline and area of residence at baseline. All statistical analyses were performed using Stata/MP V.16.0.

## 3 RESULTS

We studied 21 341 participants after excluding 886 participants who withdrew, 236 277 participants who did not report having diabetes, 2667 participants who died before 2013, 1300 participants who only dispensed acarbose and/or insulin and 4886 participants who did not have GLM records since 1 January 2013 (Figure S2). Of these participants, 43% were female, mean (SD) age in 2013 was 70.1 (9.8) years, age at diabetes diagnosis was 57.4 (11.6) years and body mass index at baseline was 30.4 (5.5) kg/m^2^. Possible drug intolerance was detected in all drug classes. Among incident users of metformin, 10.1% had only one dispensing record of the medication between 2013 and 2019. The respective figures for other drug classes were 9.8% for sulfonylurea, 5.2% for DPP‐4i, 17.4% for thiazolidinedione, 10.0% for GLP‐1RA and 6.5% for SGLT2i. Characteristics of prevalent and incident users of each drug class are available in Table S1.

### 3.1 Medication adherence in prevalent users

**FIGURE 1:**
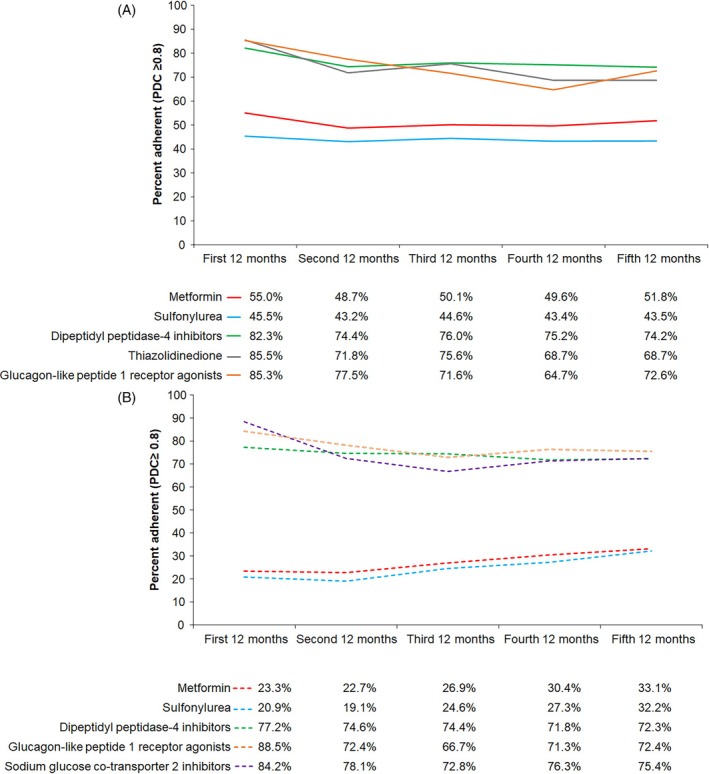
Adherence of glucose‐lowering medications over time in 5‐year continuous (a) prevalent users and (b) incident users.

There was a consistent pattern across all drug classes wherein the mean PDC was highest in the first 12 months, followed by a drop in the second 12 months and remaining relatively stable thereafter (Table 1). The mean PDC in the first 12 months ranged from 0.73 for sulfonylurea to 0.89 for thiazolidinedione. Interestingly, the 5‐year mean PDC of participants who took the same drug class for the full 5‐year period was comparable to the mean PDC in the first 12 months. Compared with mean PDCs, median PDCs were higher but showed a similar pattern for DPP‐4i, thiazolidinedione and GLP‐1RA. For metformin, the median PDCs were lower than the mean PDCs from the third 12 months. A similar pattern was observed for adherence, where the percentage of users achieving a PDC ≥0.8 was highest in the first 12 months, followed by a drop in the second 12 months. For participants who took the same drug class for the full 5‐year period, a higher percentage of adherent users compared to the first 12 months was only observed for GLP‐1RA. While the percentage of DPP‐4i users who were adherent to the medication remained relatively unchanged from the second 12 months, the percentage of adherent GLP‐1RA users declined from the peak in the first 12 months before increasing in the fifth 12 months to a level similar to the third 12 months (Figure 1A). Adherence to metformin and sulfonylurea was relatively low, with half and less than half of the users, respectively, achieving a PDC ≥0.80 during the five 12‐month periods. Nevertheless, the percentage of metformin users who switched to other drug classes was low compared to users of other drug classes (Table 1). Furthermore, adherence to metformin improved and was comparable to that for GLP‐1RA when PDC was estimated in 1717 users of metformin fixed‐dose combinations (Table S2). The results further improved when SCD of 60 was applied.

**TABLE 1:** Adherence of glucose‐lowering medication in prevalent users by drug class over five 12‐month periods and the full 5 years.

	12‐month period					Full 5 years
	First	Second	Third	Fourth	Fifth	
Metformin						
*N*	14 816	13 687	12 779	11 845	11 056	10 235
Participant included for full 12‐month analysis	92.4%	93.4%	92.7%	93.4%	92.6%	100%
Participant included for partial 12‐month analysis^a^						
Died or reached 31/12/2019	1.5%	1.6%	1.9%	1.8%	2.2%	0%
Discontinued drug class	2.3%	2.0%	2.3%	2.0%	2.2%	0%
Switched drug class	3.8%	3.0%	3.1%	2.8%	3.0%	0%
Mean (SD) analysis days	353 (51)	356 (44)	355 (46)	355 (46)	354 (47)	1826
Mean (SD) proportion of days covered	0.77 (0.22)	0.71 (0.27)	0.72 (0.27)	0.72 (0.27)	0.74 (0.26)	0.77 (0.21)
Median (IQR) proportion of days covered	0.81 (0.61–0.99)	0.74 (0.52–1.00)	0.53 (0.30–0.77)	0.55 (0.30–0.77)	0.57 (0.34–0.80)	0.79 (0.63–0.99)
Proportion of days covered ≥0.8 (i.e. adherent)	52.1%	45.2%	46.9%	47.3%	50.2%	49.0%
Sulfonylurea						
*N*	6512	5567	4776	4069	3523	3067
Participant included for full 12‐month analysis	85.4%	85.9%	85.2%	86.6%	87.0%	100%
Participant included for partial 12‐month analysis^a^						
Died or reached 31/12/2019	2.8%	2.9%	3.5%	3.3%	3.3%	0%
Discontinued drug class	1.5%	1.3%	1.6%	1.4%	4.0%	0%
Switched drug class	10.3%	9.9%	9.7%	8.7%	5.7%	0%
Mean (SD) analysis days	342 (69)	344 (65)	344 (64)	344 (66)	345 (65)	1826
Mean (SD) proportion of days covered	0.73 (0.24)	0.65 (0.30)	0.66 (0.30)	0.66 (0.30)	0.66 (0.29)	0.71 (0.24)
Median (IQR) proportion of days covered	0.74 (0.54–0.99)	0.63 (0.42–0.99)	0.66 (0.44–0.99)	0.66 (0.45–0.98)	0.66 (0.45–0.98)	0.72 (0.52–0.97)
Proportion of days covered ≥0.8 (i.e. adherent)	46.0%	40.9%	41.8%	41.5%	41.7%	41.6%
Dipeptidyl peptidase‐4 inhibitor						
*N*	2958	2599	2306	2033	1821	1573
Participant included for full 12‐month analysis	87.9%	88.7%	88.2%	89.6%	86.4%	100%
Participant included for partial 12‐month analysis^a^						
Died or reached 31/12/2019	1.2%	2.2%	2.2%	2.0%	2.6%	0%
Discontinued drug class	0.7%	0.8%	1.1%	0.9%	1.4%	0%
Switched drug class	10.2%	8.3%	8.5%	7.5%	9.6%	0%
Mean (SD) analysis days	345 (67)	345 (65)	347 (61)	349 (59)	343 (67)	1826
Mean (SD) proportion of days covered	0.88 (0.16)	0.84 (0.20)	0.84 (0.20)	0.84 (0.20)	0.84 (0.20)	0.88 (0.14)
Median (IQR) proportion of days covered	0.95 (0.84–1.00)	0.91 (0.77–0.99)	0.90 (0.78–1.00)	0.90 (0.79–0.99)	0.90 (0.78–0.99)	0.92 (0.81–0.99)
Proportion of days covered ≥0.8 (i.e. adherent)	80.6%	71.8%	72.7%	73.0%	72.2%	77.2%
Thiazolidinedione						
*N*	724	456	329	252	174	131
Participant included for full 12‐month analysis	63.0%	72.2%	76.6%	69.0%	75.3%	100%
Participant included for partial 12‐month analysis^a^						
Died, discontinued drug class, or reached 31 December 2019	1.6%	1.7%	2.1	3.2%	3.4	0%
Switched drug class	35.4%	26.1%	21.3%	27.8%	21.3%	0%
Mean (SD) analysis days	298 (104)	317 (94)	325 (89)	311 (104)	315 (104)	1826
Mean (SD) proportion of days covered	0.89 (0.13)	0.80 (0.21)	0.81 (0.21)	0.79 (0.19)	0.79 (0.21)	0.86 (0.12)
Median (IQR) proportion of days covered	0.94 (0.85–0.98)	0.88 (0.72–0.94)	0.89 (0.76–0.94)	0.85 (0.72–0.92)	0.87 (0.70–0.93)	0.89 (0.80–0.95)
Proportion of days covered ≥0.8 (i.e. adherent)	82.0%	65.6%	69.0%	63.5%	64.4%	75.6%
Glucagon‐like peptide‐1 receptor agonist						
*N*	252	191	160	127	114	102
Participant included for full 12‐month analysis	75.8%	83.8%	79.4%	89.7%	89.5%	100%
Participant included for partial 12‐month analysis^a^	24.2%	16.2%	20.6%	10.3%	10.5%	0%
Mean (SD) analysis days	326 (88)	343 (66)	327 (87)	350 (55)	344 (67)	1826
Mean (SD) proportion of days covered	0.87 (0.17)	0.80 (0.25)	0.80 (0.23)	0.79 (0.24)	0.82 (0.24)	0.87 (0.15)
Median (IQR) proportion of days covered	0.93 (0.79–1.00)	0.89 (0.71–1.00)	0.86 (0.70–1.00)	0.89 (0.68–1.00)	0.91 (0.77–1.00)	0.91 (0.82–1.00)
Proportion of days covered ≥0.8 (i.e. adherent)	74.2%	66.0%	60.6%	63.8%	71.1%	77.5%
Sodium‐glucose cotransporter‐2 inhibitor	Drug class was first dispensed in 2013					

^a^ Participants who did not have dispensing records to cover the full 12‐month period due to death, discontinuation of the drug class, switch to another drug class, or completion of the study period (31 December 2019), whichever came first, during the 12‐month period.

**FIGURE 2:**
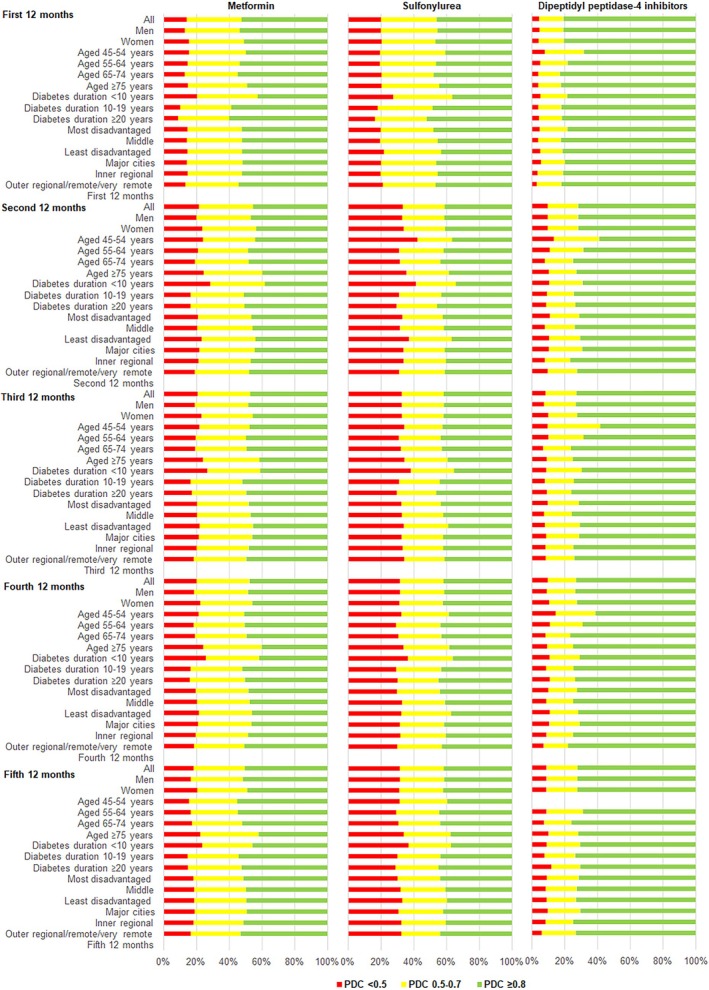
Level of adherence based on proportion of days covered (PDC) by drug class and subgroups for prevalent users.

Results by sex, age group, duration of diabetes, socio‐economic status and area of residence for metformin, sulfonylurea and DPP‐4i are presented in Table S3 and Figure 2. For other drug classes, the respective sample sizes were too small to provide meaningful results. Medication adherence differed between men and women only for metformin. Adherence to metformin was consistently lower in women compared to men over the five 12‐month periods. For the analysis by age group, compared to the youngest age group, adherence in the group ≥75 years was lower for metformin but higher for DPP‐4i. Adherence to DPP‐4i was also higher in the age 65–74 years group. Compared to the group with the duration of diabetes <10 years, the groups with longer duration of diabetes consistently had higher percentages of participants adherent to metformin and sulfonylurea. Compared to the socio‐economically most disadvantaged group, the least disadvantaged group had lower percentages of participants adherent to sulfonylurea in some periods. For analysis by area of residence, where differences exist, adherence was higher in inner regional compared to major cities. Of note, the pattern for the level of adherence within a subgroup was similar between drug classes, with sulfonylurea having higher percentages and DPP‐4i having lower percentages, of participants who were strongly nonadherent (PDC <0.5) compared to metformin (Figure 2).

### 3.2 Medication adherence in incident users

Compared with prevalent users of the same drug class, mean PDC in incident users of metformin and sulfonylurea was consistently lower and showed a U‐shaped pattern (Table 2). Mean PDC in incident users of DPP‐4i and GLP1‐RA was comparable to prevalent users and mean PDC in incident users of SGLT2i was similarly high. Like prevalent users, the 5‐year mean PDC of incident users was comparable to the mean PDC in the first 12 months. However, of those who took the same drug class for the full 5‐year period, a higher percentage of adherent users compared to the first 12 months was only observed for SGLT2i. Compared with prevalent users of the same drug class, incident users were less adherent to medication, with less than a third of metformin and sulfonylurea users achieving a PDC ≥0.80 during the five 12‐month periods (Figure 1B). Adherence to DPP‐4i, GLP1‐RA and SGLT2i was high in the first 12 months (77.2–88.5% achieved PDC ≥0.80) and up to three‐quarters of incident users remained adherent by the fifth 12‐month period (72.3–75.4% achieved PDC ≥0.8). Of note, higher percentages of GLP‐1RA incident users switched to another drug class in the first three 12‐month periods, and higher percentages of SGLT2i incident users discontinued using the drug class from the second 12‐month period onwards than incident users of other drug classes (Table 2).

**TABLE 2:** Adherence of glucose‐lowering medication in incident users by drug class over five 12‐month periods and the full 5 years.

	12‐month period					Full 5 years
	First	Second	Third	Fourth	Fifth	
Metformin						
*N*	4035	3019	2504	2047	1629	1168
Participant included for full 12‐month analysis	74.9%	83.0%	81.8%	79.6%	71.8%	100%
Participant included for partial 12‐month analysis^a^						
Died or reached 31/12/2019	4.3%	4.8%	6.6%	7.9%	13.8%	0%
Discontinued drug class	10.1%	8.0%	8.6%	9.7%	11.5%	0%
Switched drug class	10.7%	4.2%	3.0%	2.8%	2.9%	0%
Mean (SD) analysis days	317 (100)	339 (73)	336 (76)	334 (78)	320 (90)	1826
Mean (SD) proportion of days covered	0.62 (0.24)	0.51 (0.29)	0.56 (0.29)	0.58 (0.29)	0.62 (0.29)	0.62 (0.21)
Median (IQR) proportion of days covered	0.61 (0.40–0.81)	0.51 (0.30–0.72)	0.56 (0.30–0.80)	0.60 (0.32–0.84)	0.63 (0.41–0.88)	0.51 (0.45–0.77)
Proportion of days covered ≥0.8 (i.e. adherent)	26.9%	20.6%	25.3%	29.5%	32.8%	22.5%
Sulfonylurea						
*N*	3038	2099	1503	1079	745	487
Participant included for full 12‐month analysis	68.1%	71.6%	71.7%	69.0%	65.4%	100%
Participant included for partial 12‐month analysis^a^						
Died or reached 31/12/2019	6.0%	7.9%	9.2%	11.4%	11.5%	0%
Discontinued drug class	4.8%	6.8%	6.7%	7.8%	9.9%	0%
Switched drug class	21.1%	13.7%	12.4%	11.8%	13.2%	0%
Mean (SD) analysis days	312 (101)	320 (90)	320 (92)	313 (96)	308 (100)	1826
Mean (SD) proportion of days covered	0.62 (0.24)	0.49 (0.29)	0.52 (0.30)	0.54 (0.30)	0.56 (0.30)	0.59 (0.21)
Median (IQR) proportion of days covered	0.59 (0.45–0.81)	0.47 (0.27–0.69)	0.51 (0.27–0.77)	0.54 (0.30–0.80)	0.54 (0.32–0.85)	0.56 (0.44–0.73)
Proportion of days covered ≥0.8 (i.e. adherent)	26.6%	18.5%	23.6%	25.4%	29.5%	18.9%
Dipeptidyl peptidase‐4 inhibitor						
*N*	5657	4030	3015	2135	1470	918
Participant included for full 12‐month analysis	71.3%	74.8%	70.7%	68.9%	62.5%	100%
Participant included for partial 12‐month analysis^a^						
Died or reached 31/12/2019	8.1%	10.2%	12.6%	16.3%	17.9%	0%
Discontinued drug class	5.0%	5.9%	7.5%	8.3%	11.6%	0%
Switched drug class	15.6%	9.1%	9.2%	6.5%	8.0%	0%
Mean (SD) analysis days	312 (101)	323 (90)	317 (94)	312 (99)	301 (104)	1826
Mean (SD) proportion of days covered	0.88 (0.18)	0.83 (0.22)	0.83 (0.21)	0.83 (0.21)	0.82 (0.21)	0.87 (0.15)
Median (IQR) proportion of days covered	0.97 (0.83–1.00)	0.91 (0.75–0.99)	0.90 (0.75–0.99)	0.90 (0.76–0.99)	0.90 (0.75–0.99)	0.91 (0.80–0.99)
Proportion of days covered ≥0.8 (i.e. adherent)	77.7%	70.2%	70.5%	70.3%	69.4%	75.9%
Thiazolidinedione						
*N*	133	71	43	32	19	11
Participant included for full 12‐month analysis	53.4%					
Participant included for partial 12‐month analysis^a^						
Died, discontinued drug class, or reached 3 December 2019	7.5%					
Switched drug class	39.1%					
Mean (SD) analysis days	275 (120)					
Mean (SD) proportion of days covered	0.88 (0.18)					
Median (IQR) proportion of days covered	0.94 (0.85–1.00)					
Proportion of days covered ≥0.8 (i.e. adherent)	81.2%					
Glucagon‐like peptide‐1 receptor agonist						
*N*	1491	778	451	268	143	87
Participant included for full 12‐month analysis	52.2%	58.0%	59.4%	53.3%	60.8%	
Participant included for partial 12‐month analysis^a^						
Died or reached 31/12/2019	11.4%	15.1%	16.4%	26.5%	18.9%	
Discontinued drug class	5.8%	8.0%	8.9%	10.1%	8.4%	
Switched drug class	30.6%	18.9%	15.3%	10.1%	11.9%	
Mean (SD) analysis days	276 (118)	291 (109)	298 (104)	277 (119)	286 (121)	
Mean (SD) proportion of days covered	0.90 (0.17)	0.82 (0.25)	0.81 (0.24)	0.81 (0.25)	0.82 (0.23)	
Median (IQR) proportion of days covered	1.00 (0.88–1.00)	0.93 (0.73–1.00)	0.90 (0.69–1.00)	0.91 (0.71–1.00)	0.90 (0.73–1.00)	
Proportion of days covered ≥0.8 (i.e. adherent)	81.5%	66.5%	65.2%	66.8%	66.4%	
Sodium‐glucose cotransporter‐2 inhibitor						
*N*	5027	3240	2170	1134	527	114
Participant included for full 12‐month analysis	64.5%	66.9%	52.3%	46.5%	21.6%	100%
Participant included for partial 12‐month analysis^a^						
Died or reached 31/12/2019	8.5%	12.6%	22.6%	26.5%	47.1%	0%
Discontinued drug class	6.5%	9.2%	16.0%	17.8%	24.3%	0%
Switched drug class	20.5%	11.3%	9.1%	9.2%	7.0%	0%
Mean (SD) analysis days	298 (110)	311 (98)	288 (107)	271 (116)	202 (121)	1826
Mean (SD) proportion of days covered	0.88 (0.18)	0.81 (0.23)	0.81 (0.22)	0.82 (0.21)	0.81 (0.23)	0.89 (0.11)
Median (IQR) proportion of days covered	0.95 (0.83–1.00)	0.88 (0.74–0.95)	0.87 (0.74–0.95)	0.89 (0.77–0.96)	0.90 (0.73–0.98)	0.92 (0.84–0.98)
Proportion of days covered ≥0.8 (i.e. adherent)	78.4%	67.0%	66.6%	70.1%	67.9%	84.2%

^a^ Participants who did not have dispensing records to cover the full 12‐month period due to death, discontinuation of the drug class, switch to another drug class or completion of the study period (31 December 2019), whichever came first, during the 12‐month period.

**FIGURE 3:**
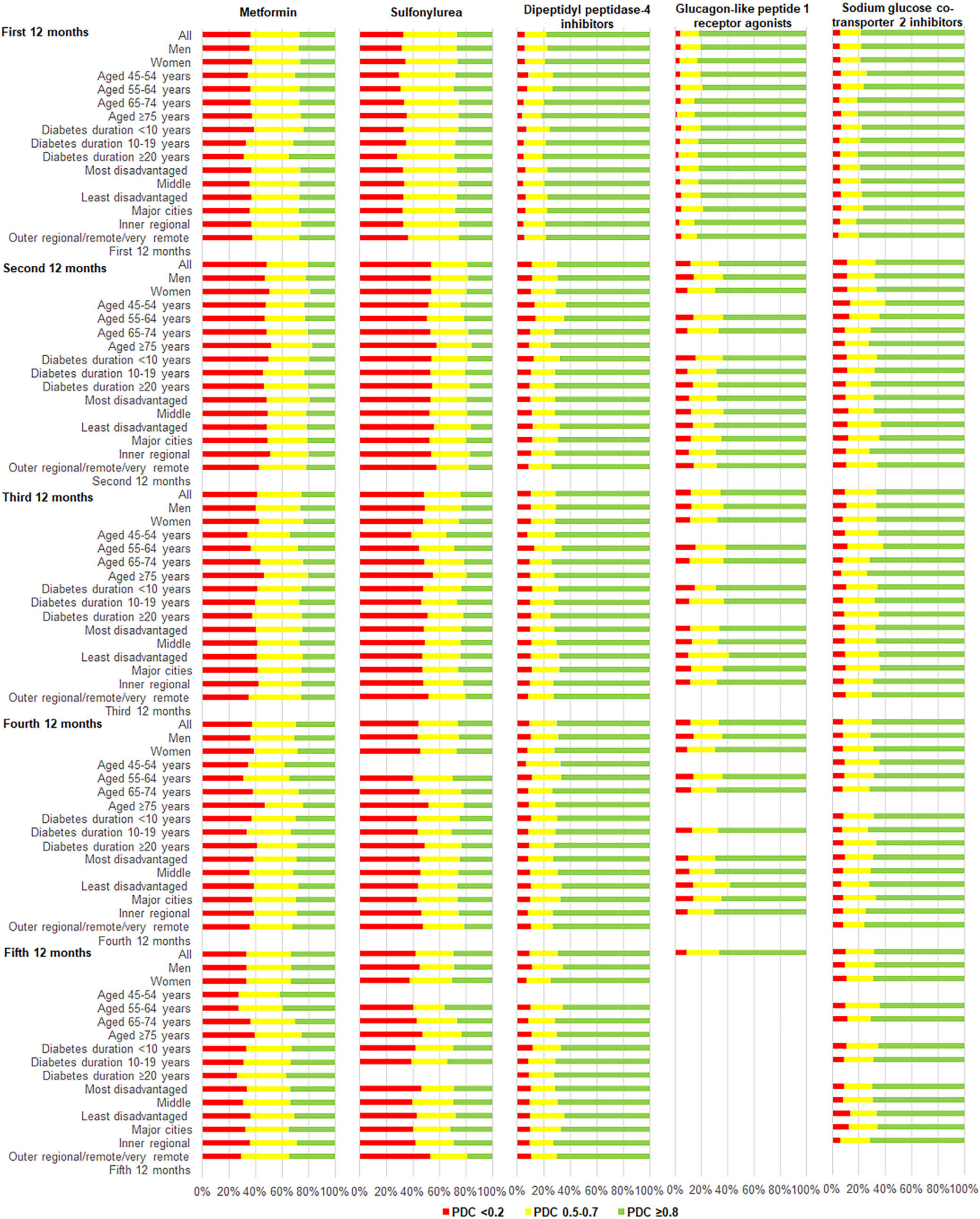
Level of adherence based on the proportion of days covered (PDC) by drug class and subgroups for incident users.

Due to small sample sizes, results for thiazolidinedione were not presented and those for GLP‐1RA and SGLT2i in later periods and for the aged 45–54 years subgroup should be interpreted with caution as reflected in the wide confidence intervals. Adherence in incident users did not differ between sexes or socio‐economic subgroups (Table S4). Compared to the youngest age group, lower percentages of incident users in the aged ≥75 years group were adherent to metformin and sulfonylurea. Adherence was generally similar between the duration of diabetes groups, with a few exceptions in users of metformin. For area of residence, where statistical significance occurred, adherence was higher in inner regional areas compared to major cities. Similar to prevalent users, the pattern for the level of adherence within a subgroup was similar between drug classes (Figure 3).

## 4 DISCUSSION

This is the first study to report the long‐term adherence of GLMs in people with diabetes in Australia. In this cohort, initial adherence to medications was good and remained relatively high over time. Lower adherence was observed in incident users compared to prevalent users for metformin and sulfonylurea. Nevertheless, no socio‐demographic subgroup was consistently more or less adherent to medications. Moreover, between 1 in 6 and 1 in 20 new GLM users discontinued, possibly due to intolerance.

Current guidelines in Australia recommend HbA1c testing at least every 6 months and medication review, which should cover the discussion of adherence and side effects, every 3 or 6 months.^14^ Nevertheless, a recent study that analysed general practice records reported that 39% of women and 34% of men with type 2 diabetes were not tested for HbA1c over the 395‐day (13‐month) period leading to the last encounter.^15^ This suggests, in practice, that medication reviews may also not have occurred as frequently as recommended. Therefore, while the adherence to GLMs in this cohort was generally good (e.g., 70% of those on DPP‐4i were adherent in the first year compared to a pooled estimate of 57% reported in a meta‐analysis of 302 911 individuals^16^; and 78% of those on SGLT2i were adherent in the first year compared to 49% reported in a meta‐analysis of 28 939 individuals),^17^ a potentially higher proportion of the cohort could achieve adherence if the recommended frequency of medication review is followed. Importantly, 5‐year mean PDC of those on the same drug class continuously for at least 5 years was comparable to the mean PDC in the first 12 months suggesting long‐term adherence is sustainable. Nevertheless, this also implied relatively lower adherence in those who discontinued or switched drug class. Adherence to metformin and sulfonylurea was substantially lower than to other GLMs. This may be explained partly by the variations in the strength and number of tablets available and the dosage required per day for metformin or sulfonylurea. After restricting the analysis to prevalent users of metformin fixed‐dose combinations and SCD of 60, adherence was comparable to that reported in England and Scotland, which used only the prescription records with daily dose recorded of general practice patients who were available for the full 12‐month analysis.^3^

While adherence to metformin and sulfonylurea was lower in incident users than prevalent users, adherence to DPP‐4i and GLP‐1RA was similarly high in both prevalent and incident users. This may be because DPP‐4i and GLP‐1RA are not commonly used as first‐line therapy.^18^ Therefore, incident users of DPP‐4i and GLP‐1RA are likely to have used other GLMs, but incident users of metformin are likely to be treatment naïve prior to starting metformin. Higher adherence to GLMs has been reported in individuals who were previously treated compared to those who were newly treated.^19^ This could also explain the higher percentage of adherent prevalent users of metformin and sulfonylurea in the groups with longer duration of diabetes than those with duration of diabetes <10 years. Moreover, compared to prevalent users of metformin and sulfonylurea, incident users of these medications will likely have started with a lower dose; hence, a packet of medications would cover more days.

Previous studies have reported that women and younger individuals with diabetes were less likely to take medication as prescribed^20^, ^21^ and that individuals with diabetes who were female, younger, poorer, had no health insurance, had no usual source of care, etc. were more likely to delay filling their scripts or to take fewer medications.^22^ Other factors related to poor adherence or non‐adherence include a negative perception of treatment, the complexity of treatment (e.g. dosing frequency as seen for metformin) and the experience of adverse side effects.^23^ Overall, our results did not suggest any subgroup to be more or less adherent to GLMs. This could be because GLMs studied here are reasonably affordable to most of the Australian population as they are subsidized by the government. The lower adherence to metformin and sulfonylurea observed in the oldest age group could be due to the recommendation of less intensive treatment in older people to reduce the risk of hypoglycaemia and minimize the use of multiple medications.^14^ Declining memory with age may also explain lower adherence in this group.

Since this study was based on dispensing records for administrative purposes, we do not have information on why a medication was discontinued or switched. Nevertheless, incident users with only one dispensing record of a medication possibly indicate intolerance to the medication. Apart from thiazolidinedione, 5–10% of incident users of other drug classes included in this study only had one dispensing record for the drug class. For thiazolidinedione, 17% of incident users only had one dispensing record. Moreover, higher proportions of both incident and prevalent users of thiazolidinedione switched to another drug class compared to users of other drug classes. This is unsurprising as thiazolidinedione is associated with serious side effects.^16^ The combined percentages for drug discontinuation and drug switch in incident users were high for the two newer drug classes, GLP‐1RA and SGLT2i. This is consistent with a Danish study, which reported the absolute 5‐year risk of discontinuing GLP‐1RA and SGLT2i was 45% and 56%, respectively.^24^ With the weight loss and cardiorenal benefits of these newer drug classes, future research into the reasons for discontinuing these medications is warranted.^25^, ^26^, ^27^, ^28^, ^29^

The strength of this research was the use of drug dispensing records to measure medication adherence in a relatively large sample of individuals with diabetes and available socio‐demographic information. Nevertheless, a few limitations warrant mention. PDC is only a proxy for measuring adherence as it measures medication on hand; we were unable to determine if the medications on hand were actually taken.^9^ Stressful life events (e.g. hospitalization and divorce) that could affect medication adherence were not considered. We were also unable to determine the proportion of participants who may have been prescribed GLMs but did not fill the scripts (primary non‐adherence) due to cost or other reasons. Of note, the 45 and Up Study was not designed to be representative of the Australian general population.^10^, ^30^ While there is no reason to assume adherence to GLMs in people with diabetes differs between jurisdictions, the higher proportions of individuals who were adherent to medications in our cohort compared to figures reported in meta‐analyses could be due to a healthy cohort effect. We also lacked biomedical data to relate medication adherence to blood glucose control.

Subsidization of medications in Australia has possibly contributed to the overall good medication adherence in this diabetes cohort. Nevertheless, adherence was still a challenge in a significant proportion of the population. Practitioners should be aware of the possibility of non‐adherence and consider this at each consultation, especially in those not meeting treatment targets.

## AUTHOR CONTRIBUTIONS

CMYL conceived the design of the study, secured funding for the study, analysed the data and drafted the manuscript. AAG, NN, SC conceived the design of the study, secured funding for the study and obtained the data. All authors contributed to the interpretation of the data and critical revision of the manuscript and approved the final version of the article to be published.

## FUNDING INFORMATION

This work was supported by a Diabetes Australia Research Program General Grant (Y20G‐LEEC). The funder had no influence in any part of the study and the researchers were independent from the funder. AAG is supported by an NHMRC Emerging Leader 1 Investigator Grant (1173784). NN is supported by the Financial Markets Foundation for Children and NHMRC Leadership Grant (APP1197940).

## CONFLICT OF INTEREST STATEMENT

The authors declare no conflicts of interest.

## PEER REVIEW

The peer review history for this article is available at https://www.webofscience.com/api/gateway/wos/peer‐review/10.1111/dom.16408.

## ETHICS STATEMENT

The 45 and Up Study was approved by the University of NSW Human Research Ethics Committee and the use of linked data for this study was approved by the NSW Population and Health Services Research Ethics Committee.

## Supporting information

Supporting Information.

## Data Availability

The data that support the findings of this study are available from the Sax Institute, but restrictions apply to the availability of these data, which were used under licence for the current study and so are not publicly available. Data are, however, available from the authors upon reasonable request and with permission of the Sax Institute (http://www.saxinstitute.org.au/).
