# Supplementary material for: Long‐term adherence to glucose‐lowering medications in adults with diabetes: A data linkage study
Source: Diabetes Obes Metab. 2025 Apr 23;27(7):3809–20. doi: 10.1111/dom.16408 (PMC12146459; doi:10.1111/dom.16408)
Supplement: Supplementary file 1 — Supporting Information. [file DOM-27-3809-s001.pdf]

Long-term adherence to glucose lowering medications in adults with diabetes: a data linkage study

Crystal Man Ying Lee, Alice A Gibson, Natasha Nassar, Stephen Colagiuri

Figure S1: Flow diagram of participant inclusion for analysis

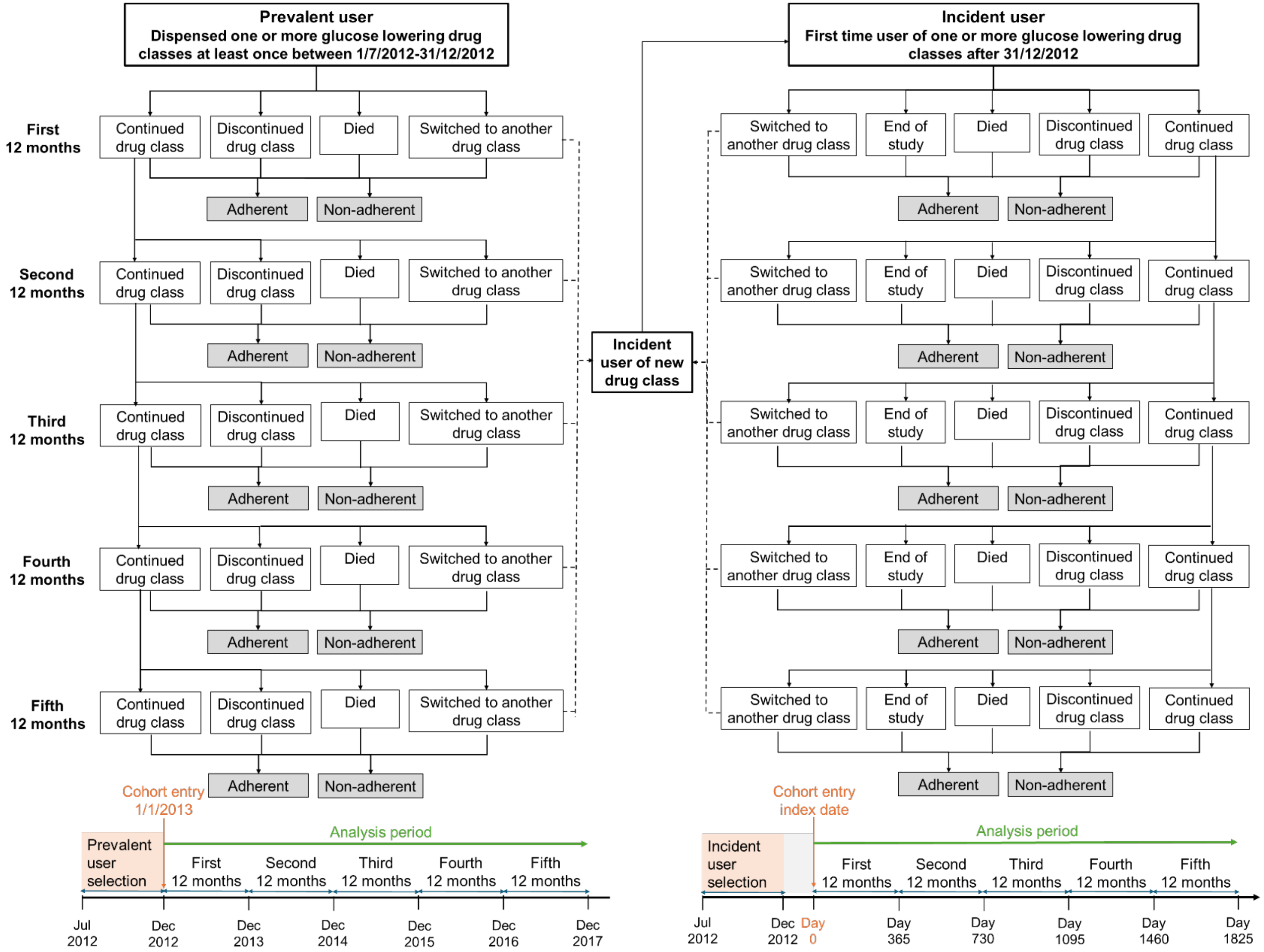

Figure S2: Flow diagram of participant selection

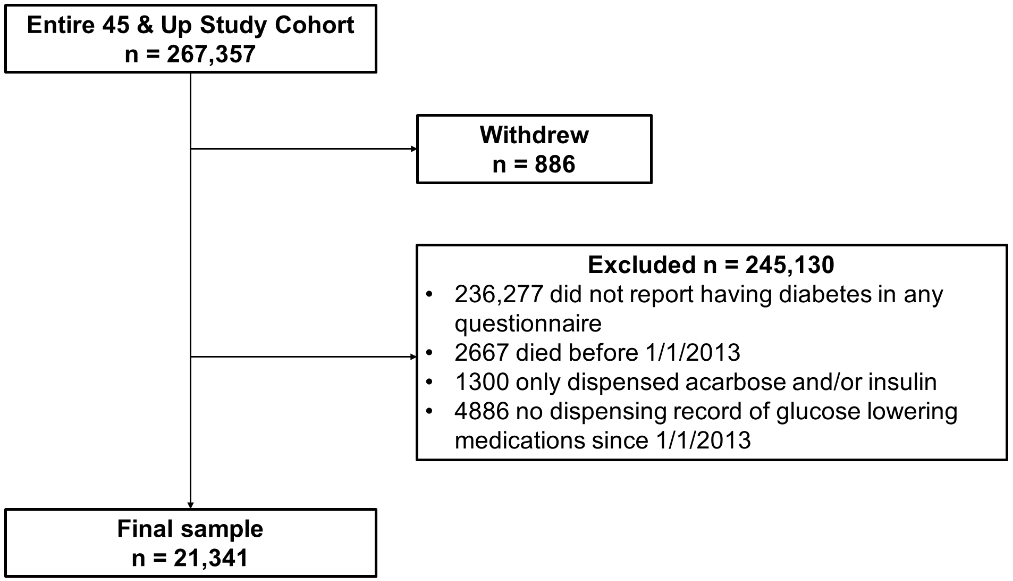

Table S1: Characteristics of participants included in medication adherence analysis by glucose lowering medication user type

Drug class	Metformin		Sulfonylurea		DPP-4i		TZD		GLP-1 RA		SGLT2i	
**User type**	**P**	**I**	**P**	**I**	**P**	**I**	**P**	**I**	**P**	**I**	**P**	**I**
**N**	14816	4035	6512	3038	2958	5657	724	133	252	1491	-	5027
**Female**	42.4%	44.8%	39.1%	44.0%	41.4%	42.9%	34.0%	46.6%	52.0%	51.2%	-	41.4%
**Mean (SD) age†**	69.7	68.3	71.7	68.5	69.3	68.7	69.7	68.0	64.7	63.7	-	64.9
	(9.4)	(9.8)	(9.9)	(9.7)	(9.3)	(9.3)	(9.4)	(8.9)	(6.9)	(7.2)		(7.6)
**Age group†**												
45–54 years	4.7%	6.9%	3.2%	6.8%	4.9%	5.5%	3.9%	5.3%	6.8%	9.8%	-	8.3%
55–64 years	26.7%	31.4%	22.5%	29.7%	27.5%	30.1%	26.9%	30.8%	42.5%	46.6%	-	41.1%
65–74 years	38.2%	36.0%	35.8%	36.7%	38.7%	37.8%	38.3%	39.1%	42.5%	35.8%	-	39.6%
≥75 years	30.5%	25.8%	38.6%	26.9%	28.9%	26.7%	30.9%	24.8%	8.3%	7.9%	-	11.1%
**Mean (SD) age at diabetes diagnosis**	56.6	59.0	56.7	57.3	55.9	56.3	53.3	54.4	50.0	50.9	-	52.4
	(11.0)	(11.8)	(11.0)	(11.0)	(10.1)	(10.8)	(10.0)	(11.3)	(8.7)	(9.2)		(10.0)
**Duration of diabetes†**												
<10 years	38.8%	62.6%	25.9%	49.9%	34.4%	42.9%	16.8%	30.2%	25.1%	37.6%	-	39.8%
10–19 years	44.9%	26.7%	53.3%	38.8%	50.7%	42.1%	58.3%	46.6%	53.2%	47.4%	-	45.2%
≥20 years	16.4%	10.7%	20.8%	11.3%	14.9%	15.0%	24.9%	23.3%	21.7%	15.0%	-	15.0%
**Mean (SD) body mass index†**	30.6	30.2	30.1	30.7	30.8	30.6	30.7	30.4	34.5	33.8	-	31.7
	(5.6)	(5.4)	(5.5)	(5.6)	(5.5)	(5.5)	(5.4)	(5.5)	(5.9)	(5.8)		(5.6)
**Area of residence†**												
Major cities	53.8%	53.3%	58.0%	53.2%	52.9%	53.5%	57.0%	41.1%	64.8%	50.1%	-	55.0%
Inner regional	34.3%	33.1%	30.3%	33.9%	35.8%	34.0%	35.1%	40.3%	24.3%	35.7%	-	32.8%

Outer regional/remote/very remote	11.9%	13.6%	11.7%	12.9%	11.3%	12.5%	7.9%	18.6%	10.9%	14.2%	-	12.2%
**Education†**												
No school certificate or other qualification	17.6%	14.5%	19.8%	16.6%	18.5%	18.1%	19.5%	22.1%	12.0%	17.9%	-	16.3%
School/higher school certificate	33.8%	34.0%	33.8%	33.6%	33.2%	33.9%	34.9%	32.8%	38.0%	33.7%	-	32.2%
Trade/apprenticeship/diploma	31.5%	33.2%	31.2%	33.4%	32.2%	31.6%	32.5%	29.0%	29.6%	31.4%	-	33.2%
University degree or higher	17.1%	18.3%	15.4%	16.4%	16.2%	16.4%	13.1%	16.0%	20.4%	17.1%	-	18.5%
**Socioeconomic status (SEIFA-IRSD)†**												
Most disadvantaged (deciles 1–3)	40.2%	37.7%	41.5%	38.0%	39.7%	40.4%	45.8%	42.6%	35.2%	40.6%	-	37.9%
Middle (deciles 4–7)	37.5%	39.6%	37.8%	38.5%	38.6%	38.5%	37.8%	42.6%	38.1%	38.7%	-	39.0%
Least disadvantaged (deciles 8–10)	22.3%	22.7%	20.7%	23.6%	21.7%	21.1%	16.4%	14.7%	26.7%	20.8%	-	23.1%
**Income (per year)†**												
<$30,000	41.9%	36.6%	46.1%	39.0%	41.5%	40.2%	45.9%	44.0%	33.1%	34.2%	-	32.8%
$30,000–69,999	24.7%	26.5%	22.2%	26.2%	24.3%	25.3%	23.4%	16.8%	24.9%	24.5%	-	28.8%
≥$70,000	16.0%	19.6%	13.3%	18.0%	16.2%	16.4%	12.7%	15.2%	24.5%	22.9%	-	21.8%
Rather not say	17.4%	17.2%	18.3%	16.8%	18.1%	18.2%	18.0%	24.0%	17.6%	18.5%	-	16.6%

DPP-4i = dipeptidyl peptidase-4 inhibitor; TZD = thiazolidinedione; GLP-1 RA = glucagon-like peptide-1 receptor agonist; SGLT2i = sodium-glucose cotransporter-2 inhibitor; P = prevalent user; I = incident user; SEIFA-IRSD = Socio-Economic Indexes for Areas- Index of Relative Socio-Economic Disadvantage;

† All socio-demographic factors measured at baseline except for age and duration of diabetes determined in 2013; (https://www.saxinstitute.org.au/solutions/45-and-up-study/use-the-45-and-up-study/data-and-technical-information/)

Table S2: Adherence of metformin in prevalent users by metformin type over five 12-month periods

	First 12 months	Second 12 months	Third 12 months	Fourth 12 months	Fifth 12 months
**Metformin alone (standard coverage days = 37 [5])**					
N	13225	11707	10364	9084	7823
Participant included for full 12-month analysis	88.4%	88.5%	87.7%	86.2%	85.8%
Participant included for partial 12-month analysis†					
Participants who died or reached 31st December 2019	1.7%	1.8%	2.1%	2.0%	2.5%
Participants who discontinued the drug class	2.5%	2.2%	2.6%	2.4%	2.6%
Participants who switched to another drug class	7.4%	7.5%	7.6%	9.4%	9.1%
Mean (SD) analysis days	347 (62)	348 (58)	348 (59)	345 (63)	344 (65)
Mean (SD) proportion of days covered	0.75 (0.23)	0.68 (0.28)	0.68 (0.28)	0.68 (0.28)	0.68 (0.27)
Median (IQR) proportion of days covered	0.79 (0.61-0.99)	0.71 (0.50-1.00)	0.71 (0.50-1.00)	0.71 (0.50-0.98)	0.71 (0.51-0.96)
Proportion of days covered ≥0.8 (i.e. adherent)	48.3%	39.2%	39.5%	38.6%	39.0%
**Metformin fixed dose combination**					
N	1717	1483	1329	1175	1073
Participant included for full 12-month analysis	85.8%	89.6%	88.4%	91.3%	89.3%
Participant included for partial 12-month analysis†					
Participants who died or reached 31st December 2019	1.6%	1.2%	1.4%	1.4%	2.1%
Participants who discontinued the drug class	1.0%	0.7%	1.1%	0.6%	1.3%
Participants who switched to another drug class	11.6%	8.5%	9.1%	6.7%	7.3%
Mean (SD) analysis days	344 (68)	348 (62)	346 (63)	352 (51)	349 (57)
Mean (SD) proportion of days covered	0.86 (0.17)	0.82 (0.22)	0.82 (0.22)	0.82 (0.22)	0.83 (0.21)
Median (IQR) proportion of days covered	0.94 (0.81-1.00)	0.90 (0.71-0.99)	0.90 (0.73-1.00)	0.90 (0.74-1.00)	0.90 (0.74-0.99)

Proportion of days covered ≥0.8 (i.e. adherent)	75.8%	67.3%	69.1%	69.6%	69.3%
**Metformin alone (standard coverage days = 60)**					
N	13225	11707	10364	9084	7823
Participant included for full 12-month analysis	88.5%	88.5%	87.6%	86.1%	85.8%
Participant included for partial 12-month analysis†					
Participants who died or reached 31st December 2019	1.7%	1.9%	2.2%	2.1%	2.6%
Participants who discontinued the drug class	2.5%	2.2%	2.7%	2.4%	2.6%
Participants who switched to another drug class	7.3%	7.4%	7.5%	9.4%	9.0%
Mean (SD) analysis days	350 (56)	351 (52)	350 (53)	348 (56)	347 (58)
Mean (SD) proportion of days covered	0.90 (0.17)	0.87 (0.23)	0.87 (0.22)	0.87 (0.22)	0.88 (0.21)
Median (IQR) proportion of days covered	1.00 (0.88-1.00)	1.00 (0.82-1.00)	1.00 (0.82-1.00)	1.00 (0.82-1.00)	1.00 (0.82-1.00)
Proportion of days covered ≥0.8 (i.e. adherent)	80.4%	76.6%	77.0%	77.1%	77.6%

†Participants who did not have dispensing records to cover the full 12-month period due to death, discontinuation of the drug class, switch to another drug class, or completion of study period (31st December 2019), whichever came first, during the 12-month period.

Table S3: Percentage of adherent (i.e., proportion of days covered ≥0.8) prevalent users for each drug class over five 12-month periods by sociodemographic factors

	First 12 months		Second 12 months		Third 12 months		Fourth 12 months		Fifth 12 months	
	Percent adherent† (95% CI)	p-value	Percent adherent† (95% CI)	p-value	Percent adherent† (95% CI)	p-value	Percent adherent† (95% CI)	p-value	Percent adherent† (95% CI)	p-value
**Metformin**										
N	13094		12124		11330		10521		9845	
Sex										
Men	53.0 (51.9, 54.1)		46.9 (45.7. 48.0)		48.2 (47.0, 49.4)		48.9 (47.6, 50.1)		52.0 (50.7, 53.3)	
Women	51.4 (50.1, 52.7)	0.067	44.1 (42.7, 45.4)	0.002	45.8 (44.3, 47.2)	0.009	46.1 (44.6, 47.6)	0.004	48.7 (47.1, 50.2)	0.001
Age										
45-54 years	52.0 (48.2, 55.8)		46.1 (42.1, 50.1)		48.3 (44.2, 52.4)		52.1 (47.9, 56.3)		56.0 (51.8, 60.2)	
55-64 years	55.4 (53.8, 57.0)	0.109	49.9 (48.3, 51.6)	0.083	50.5 (48.7, 52.2)	0.336	51.7 (49.9, 53.4)	0.848	55.7 (53.9, 57.4)	0.885
65-74 years	54.2 (52.8, 55.5)	0.298	47.8 (46.3, 49.2)	0.438	49.0 (47.5, 50.5)	0.744	49.3 (47.8, 50.8)	0.221	51.5 (50.0, 53.1)	0.055
≥75 years	47.2 (45.7, 48.8)	0.023	38.8 (37.2, 40.5)	0.001	41.0 (39.3, 42.7)	0.001	39.9 (38.1, 41.8)	<0.001	41.7 (39.7, 43.6)	<0.001
Duration of diabetes										
<10 years	41.6 (40.2, 42.9)		37.0 (35.7, 38.4)		40.0 (38.5, 41.4)		40.6 (39.1, 42.1)		44.8 (43.2, 46.4)	
10-19 years	58.6 (57.3, 59.8)	<0.001	50.8 (49.5, 52.1)	<0.001	52.1 (50.8, 53.5)	<0.001	52.3 (50.9, 53.7)	<0.001	54.3 (52.9, 55.8)	<0.001
≥20 years	60.7 (58.6, 62.8)	<0.001	52.3 (50.1, 54.6)	<0.001	50.9 (48.5, 53.2)	<0.001	52.4 (49.9, 54.8)	<0.001	54.7 (52.1, 57.2)	<0.001
Socioeconomic status										
Most disadvantaged	51.8 (50.4, 53.2)		46.1 (44.7, 47.6)		47.8 (46.4, 49.3)		47.9 (46.4, 49.5)		51.0 (49.4, 52.6)	
Middle	52.2 (50.9, 53.6)	0.656	45.5 (44.1, 47.0)	0.553	47.0 (45.5, 48.5)	0.428	47.7 (46.2, 49.3)	0.836	50.1 (48.5, 51.7)	0.455

Least disadvantaged	53.3 (51.5, 55.2)	0.191	45.1 (43.2, 47.0)	0.420	46.3 (44.4, 48.3)	0.240	47.3 (45.3, 49.3)	0.626	50.8 (48.7, 52.9)	0.874
Area of residence										
Major cities	51.7 (50.6, 52.9)		44.6 (43.4, 45.8)		46.2 (44.9, 47.5)		46.4 (45.1, 47.7)		49.5 (48.2, 50.9)	
Inner regional	52.6 (51.2, 54.1)	0.358	46.6 (45.1, 48.1)	0.043	48.0 (46.4, 49.6)	0.080	48.7 (47.1, 50.3)	0.035	51.6 (49.9, 53.2)	0.068
Outer regional/ remote/very remote	54.0 (51.5, 56.4)	0.113	47.8 (45.2, 50.4)	0.031	49.2 (46.5, 51.8)	0.054	50.7 (47.9, 53.5)	0.008	52.7 (49.8, 55.6)	0.054
**Sulfonylurea**										
N	5788		4962		4250		3642		3163	
Sex										
Men	45.3 (43.7, 47.0)		41.2 (39.4, 42.9)		41.7 (39.8, 43.6)		41.1 (39.0, 43.1)		41.6 (39.4, 43.8)	
Women	46.6 (44.6, 48.7)	0.337	41.0 (38.8, 43.2)	0.910	42.1 (39.8, 44.5)	0.794	42.3 (39.7, 44.9)	0.474	42.1 (39.4, 44.9)	0.774
Age										
45-54 years	43.6 (36.6, 50.7)		39.1 (31.5, 46.6)		44.4 (35.9, 52.9)		40.3 (31.1, 49.5)		40.2 (30.2, 50.3)	
55-64 years	48.4 (45.7, 51.1)	0.213	43.1 (40.2, 46.0)	0.331	45.0 (41.9, 48.1)	0.893	44.7 (41.4, 48.0)	0.382	45.6 (42.1, 49.2)	0.325
65-74 years	47.9 (45.8, 50.1)	0.253	43.7 (41.4, 46.0)	0.258	42.7 (40.3, 45.1)	0.706	43.0 (40.4, 45.6)	0.581	44.0 (41.3, 46.8)	0.479
≥75 years	42.5 (40.4, 44.6)	0.763	37.5 (35.3, 39.7)	0.692	38.8 (36.3, 41.2)	0.207	37.9 (35.2, 40.6)	0.618	36.4 (33.5, 39.4)	0.470
Duration of diabetes										
<10 years	35.6 (33.1, 38.0)		33.3 (30.7, 35.9)		34.4 (31.6, 37.2)		35.2 (32.2, 38.2)		36.0 (32.8, 39.2)	
10-19 years	48.1 (46.4, 49.9)	<0.001	42.8 (40.9, 44.7)	<0.001	43.8 (41.8, 45.8)	<0.001	43.3 (41.1, 45.5)	<0.001	43.5 (41.2, 45.9)	<0.001
≥20 years	52.8 (49.9, 44.7)	<0.001	46.6 (43.5, 49.7)	<0.001	46.8 (43.4, 50.2)	<0.001	45.7 (42.0, 49.5)	<0.001	45.4 (41.3, 49.4)	<0.001
Socioeconomic status										
Most disadvantaged	47.5 (45.5, 49.6)		42.2 (40.0, 44.4)		43.3 (40.9, 45.6)		43.9 (41.3, 46.5)		43.6 (40.8, 46.4)	

Middle	45.3 (43.2, 47.3)	0.121	42.0 (39.7, 44.2)	0.875	41.7 (39.3, 44.1)	0.363	41.3 (38.8, 43.9)	0.172	40.9 (38.2, 43.7)	0.189
Least disadvantaged	43.6 (40.7, 46.4)	0.028	37.4 (34.4, 40.4)	0.013	39.6 (36.4, 42.8)	0.079	37.8 (34.3, 41.2)	0.007	40.2 (36.5, 43.8)	0.158
Area of residence										
Major cities	46.5 (44.8, 48.2)		41.4 (39.6, 43.2)		42.1 (40.1, 44.0)		41.8 (39.7, 44.0)		42.1 (39.8, 44.4)	
Inner regional	44.5 (42.2, 46.9)	0.180	40.6 (38.1, 43.1)	0.621	42.6 (39.4, 44.9)	0.956	41.0 (38.0, 43.9)	0.645	40.9 (37.7, 44.0)	0.548
Outer regional/ remote/very remote	45.7 (41.9, 49.5)	0.690	40.7 (36.8, 44.7)	0.757	40.3 (36.0, 44.6)	0.474	41.8 (37.2, 46.4)	0.996	42.6 (37.6, 47.6)	0.854
**Dipeptidyl peptidase-4 inhibitor**										
N	2640		2323		2063		1821		1626	
Sex										
Men	80.2 (78.2, 82.1)		72.4 (70.1, 74.9)		72.8 (70.3, 75.3)		73.2 (70.6, 75.9)		71.7 (68.8, 74.6)	
Women	81.0 (78.7, 83.4)	0.570	72.4 (69.5, 75.2)	0.972	73.0 (70.0, 76.0)	0.903	72.3 (60.1, 75.4)	0.653	72.6 (69.3, 76.0)	0.683
Age										
45-54 years	68.2 (60.3, 76.2)		58.9 (49.8, 67.9)		60.3 (50.8, 69.7)		50.1 (49.1, 69.0)		63.0 (52.7, 73.4)	
55-64 years	79.0 (76.0, 82.0)	0.006	70.0 (66.4, 73.5)	0.019	69.4 (65.5, 73.1)	0.070	69.6 (65.6, 73.7)	0.045	69.5 (65.2, 73.8)	0.244
65-74 years	82.1 (79.7, 845)	<0.001	74.7 (71.9, 77.6)	<0.001	75.6 (72.7, 78.6)	0.001	75.2 (72.0, 78.4)	0.001	75.5 (72.2, 78.9)	0.016
≥75 years	82.1 (79.3, 84.9)	<0.001	74.0 (70.6, 77.4)	0.001	74.8 (71.2, 78.4)	0.003	75.2 (71.4, 79.0)	0.002	71.6 (67.3, 75.8)	0.124
Duration of diabetes										
<10 years	79.0 (76.3, 81.6)		69.7 (66.5, 72.9)		70.6 (67.2, 73.9)		71.5 (68.0, 75.0)		71.2 (67.5, 75.0)	
10-19 years	81.6 (79.5, 83.4)	0.127	74.2 (71.2, 76.7)	0.030	74.0 (71.3, 76.6)	0.111	73.9 (71.1, 76.7)	0.291	73.3 (70.3, 76.3)	0.409
≥20 years	80.6 (76.5, 84.6)	0.528	73.0 (68.1, 77.8)	0.288	74.6 (69.5, 79.7)	0.211	72.1 (66.3, 77.8)	0.879	69.8 (63.6, 76.0)	0.701
Socioeconomic status										

Most disadvantaged	78.1 (75.5, 80.6)		71.1 (68.1, 74.0)		71.0 (67.7, 74.2)		71.7 (68.3, 75.1)		70.2 (66.5, 73.9)	
Middle	82.6 (80.3, 84.9)	0.010	73.9 (71.0, 76.7)	0.185	75.4 (72.4, 78.3)	0.049	74.2 (71.0, 77.4)	0.294	72.5 (69.0, 75.9)	0.378
Least disadvantaged	81.3 (78.1, 84.5)	0.135	72.3 (68.4, 76.2)	0.623	71.8 (67.7, 75.9)	0.761	72.3 (68.0, 76.7)	0.817	74.3 (69.9, 78.8)	0.174
Area of residence										
Major cities	79.8 (77.7, 82.0)		69.8 (67.2, 72.4)		71.0 (68.2, 73.7)		70.7 (67.7, 73.6)		69.9 (66.7, 73.0)	
Inner regional	81.1 (78.5, 83.6)	0.466	76.1 (73.2, 79.0)	0.002	74.9 (71.7, 78.0)	0.075	74.5 (71.1, 77.8)	0.100	75.1 (71.5, 78.6)	0.035
Outer regional/ remote/very remote	82.1 (77.8, 86.4)	0.367	72.9 (67.5, 78.2)	0.335	75.0 (69.4, 80.7)	0.223	77.8 (72.0, 83.7)	0.046	73.0 (66.4, 79.6)	0.413

†Adjusted for sex, age in 2013, duration of diabetes in 2013, socioeconomic status at baseline and area of residence at baseline

Table S4: Percentage of adherent (i.e., proportion of days covered ≥0.8) incident users for each drug class over five 12-month periods by sociodemographic factors

	First 12 months		Second 12 months		Third 12 months		Fourth 12 months		Fifth 12 months	
	Percent adherent† (95% CI)	p-value	Percent adherent† (95% CI)	p-value	Percent adherent† (95% CI)	p-value	Percent adherent† (95% CI)	p-value	Percent adherent† (95% CI)	p-value
**Metformin**										
N	3539		2658		2210		1812		1452	
Sex										
Men	27.9 (25.9, 29.9)		22.5 (20.4, 24.6)		27.0 (24.5, 29.5)		31.6 (28.8, 34.5)		34.0 (30.8, 37.3)	
Women	25.7 (23.6, 27.9)	0.140	18.5 (16.2, 20.7)	0.011	24.2 (21.6, 26.9)	0.140	27.9 (24.8, 31.0)	0.085	33.2 (29.6, 36.8)	0.737
Age										
45-54 years	31.2 (25.6, 36.9)		23.1 (17.1, 29.2)		35.5 (28.0, 42.9)		37.6 (29.4, 45.8)		39.5 (30.3, 48.8)	
55-64 years	27.4 (24.7, 30.0)	0.215	23.3 (20.5, 26.2)	0.958	28.6 (25.3, 31.9)	0.085	35.4 (31.6, 39.2)	0.639	40.1 (35.8, 44.5)	0.907
65-74 years	27.3 (24.9, 29.8)	0.24	20.6 (18.0, 23.1)	0.428	24.5 (21.5, 27.4)	0.004	26.5 (23.2, 29.8)	0.010	31.2 (27.4, 35.0)	0.093
≥75 years	24.6 (21.8, 27.5)	0.036	16.5 (13.6, 19.5)	0.042	20.7 (17.0, 24.4)	<0.001	24.4 (20.0, 28.9)	0.004	24.9 (19.7, 30.0)	0.005
Duration of diabetes										
<10 years	23.5 (21.7, 25.2)		19.1 (17.3, 21.0)		24.7 (22.5, 26.8)		28.4 (25.9, 30.9)		32.5 (29.6, 35.4)	
10-19 years	31.9 (28.9, 34.9)	<0.001	24.4 (21.1, 27.7)	0.005	28.5 (24.6, 32.4)	0.089	34.3 (29.8, 38.9)	0.024	35.3 (30.2, 40.4)	0.352
≥20 years	35.1 (30.2, 40.0)	<0.001	22.3 (16.8, 27.7)	0.263	27.6 (20.9, 34.3)	0.402	30.5 (23.0, 37.9)	0.610	39.6 (30.4, 48.8)	0.140
Socioeconomic status										
Most disadvantaged	26.6 (24.2, 29.0)		19.1 (16.6, 21.6)		24.9 (21.9, 27.9)		28.8 (25.3, 32.2)		34.8 (30.7, 38.9)	
Middle	27.0 (24.7, 29.3)	0.845	21.9 (19.4, 24.4)	0.114	27.1 (24.2, 30.0)	0.303	32.0 (28.6, 35.3)	0.193	35.0 (31.2, 38.9)	0.946

Least disadvantaged	27.3 (24.2, 30.5)	0.733	21.2 (17.8, 24.6)	0.326	24.8 (21.0, 28.7)	0.974	28.4 (23.9, 32.8)	0.894	29.6 (24.7, 34.5)	0.122
Area of residence										
Major cities	27.6 (25.6, 29.6)		20.6 (18.5, 22.7)		25.3 (22.8, 27.8)		29.5 (26.6, 32.4)		35.5 (32.0, 38.9)	
Inner regional	26.0 (23.4, 28.5)	0.329	20.1 (17.4, 22.8)	0.761	26.2 (22.9, 29.5)	0.669	29.5 (25.8, 33.3)	0.997	30.0 (25.8, 34.3)	0.058
Outer regional/ remote/very remote	26.6 (22.6, 30.6)	0.670	22.5 (18.1, 26.9)	0.440	26.8 (21.8, 31.8)	0.598	32.5 (26.8, 38.2)	0.365	34.9 (28.6, 41.2)	0.884
**Sulfonylurea**										
N	2695		1877		1335		955		653	
Sex										
Men	26.8 (24.5, 29.0)		18.7 (16.4, 21.1)		23.1 (20.1, 26.0)		25.2 (21.7, 28.8)		29.1 (24.7, 33.6)	
Women	26.4 (23.9, 28.9)	0.818	19.0 (16.4, 21.7)	0.864	24.9 (21.3, 28.4)	0.443	27.5 (23.1, 31.9)	0.430	30.9 (25.4, 36.4)	0.624
Age										
45-54 years	28.3 (21.9, 34.8)		23.3 (15.9, 30.6)		32.9 (23.4, 42.5)		37.0 (24.7, 49.3)		37.3 (22.2, 52.3)	
55-64 years	29.4 (26.3, 32.6)	0.761	22.3 (18.7, 25.8)	0.802	29.2 (24.7, 33.7)	0.478	30.3 (25.0, 35.7)	0.310	36.2 (29.6, 42.8)	0.897
65-74 years	25.7 (23.0, 28.4)	0.456	18.2 (15.3, 21.0)	0.178	21.3 (17.8, 24.8)	0.015	24.0 (19.7, 28.3)	0.035	26.8 (21.4, 32.1)	0.176
≥75 years	24.1 (20.9, 27.3)	0.237	14.9 (11.7, 18.1)	0.027	18.8 (14.5, 23.1)	0.005	21.9 (16.6, 27.3)	0.019	24.8 (17.9, 31.7)	0.121
Duration of diabetes										
<10 years	24.9 (22.6, 27.2)		18.0 (15.6, 20.5)		22.0 (18.9, 25.2)		23.3 (19.5, 27.1)		27.9 (23.0, 32.8)	
10-19 years	28.1 (25.4, 30.8)	0.079	20.3 (17.4, 23.2)	0.242	26.4 (22.7, 30.1)	0.083	30.2 (25.7, 34.7)	0.023	33.4 (27.8, 39.0)	0.150
≥20 years	29.0 (23.8, 34.2)	0.150	17.3 (12.0, 22.5)	0.795	22.5 (15.5, 29.4)	0.913	23.7 (15.0, 32.3)	0.942	24.9 (14.4, 35.3)	0.616
Socioeconomic status										
Most disadvantaged	27.3 (24.5, 30.1)		20.2 (17.1, 23.2)		23.2 (19.4, 27.0)		23.8 (19.2, 28.4)		28.8 (22.7, 34.8)	

Middle	25.9 (23.2, 28.6)	0.478	19.5 (16.6, 22.4)	0.748	24.6 (20.9, 28.3)	0.611	26.9 (22.5, 31.4)	0.341	32.3 (26.8, 37.9)	0.398
Least disadvantaged	26.7 (23.2, 30.2)	0.803	16.0 (12.6, 19.4)	0.089	23.5 (18.9, 28.1)	0.924	28.2 (22.4, 33.9)	0.256	27.4 (30.7, 34.2)	0.784
Area of residence										
Major cities	28.1 (25.7, 30.5)		20.5 (17.9, 23.4)		25.1 (21.9, 28.3)		26.6 (22.8, 30.4)		32.3 (27.5, 37.2)	
Inner regional	24.8 (22.0, 27.7)	0.092	17.4 (14.5, 20.4)	0.143	23.1 (19.0, 27.2)	0.466	27.0 (21.9, 32.2)	0.891	30.5 (23.9, 37.1)	0.672
Outer regional/ remote/very remote	25.1 (20.5, 29.7)	0.273	16.3 (11.8, 20.8)	0.143	20.2 (14.3, 36.0)	0.174	22.6 (15.3, 29.9)	0363	18.6 (10.6, 26.6)	0.013
**Dipeptidyl peptidase-4 inhibitor**										
N	5022		3587		2683		1898		1313	
Sex										
Men	76.2 (74.6, 77.7)		69.3 (67.3, 71.3)		69.9 (67.6, 62.1)		69.1 (66.4, 71.7)		65.9 (62.6, 69.2)	
Women	78.9 (77.2, 80.6)	0.022	71.0 (68.7, 73.3)	0.267	71.6 (68.9, 74.2)	0.350	71.9 (68.7, 75.1)	0.194	74.7 (71.0, 78.5)	0.001
Age										
45-54 years	73.4 (68.3, 78.6)		64.4 (57.6, 71.1)		72.4 (65.0, 79.8)		68.8 (59.8, 77.8)		71.5 (61.1, 81.8)	
55-64 years	73.4 (71.2, 75.6)	0.989	65.6 (62.7, 68.4)	0.744	66.3 (63.1, 69.5)	0.156	66.4 (62.6, 70.2)	0.638	65.7 (61.1, 70.3)	0.334
65-74 years	79.1 (77.3, 80.9)	0.032	71.7 (69.4, 74.1)	0.035	73.5 (70.9, 76.2)	0.779	72.1 (69.0, 75.3)	0.480	71.4 (67.6, 75.2)	0.987
≥75 years	80.5 (78.2, 82.7)	0.010	74.4 (71.5, 77.4)	0.005	70.9 (67.3, 74.5)	0.726	72.2 (67.9, 76.5)	0.500	70.5 (65.2, 75.9)	0.877
Duration of diabetes										
<10 years	76.0 (74.2, 77.8)		68.8 (66.5, 71.1)		68.6 (65.9, 71.3)		69.5 (66.4, 72.7)		67.6 (63.8, 71.4)	
10-19 years	78.0 (76.2, 79.7)	0.136	71.3 (69.0, 73.6)	0.142	71.4 (68.8, 74.0)	0.143	70.4 (67.3, 73.6)	0.685	70.8 (67.1, 74.6)	0.239
≥20 years	79.7 (76.7, 82.6)	0.047	70.2 (66.0, 74.3)	0.582	74.2 (69.6, 78.9)	0.047	71.6 (65.9, 77.3)	0.539	71.1 (64.0, 78.2)	0.409
Socioeconomic status										

Most disadvantaged	75.7 (73.8, 77.6)		70.0 (67.6, 72.4)		70.9 (68.2, 73.7)		72.2 (69.0, 75.4)		71.0 (67.2, 74.9)	
Middle	79.2 (77.4, 81.0)	0.010	70.7 (68.3, 73.1)	0.659	70.4 (67.6, 73.2)	0.803	69.6 (66.2, 73.0)	0.279	70.0 (66.0, 74.1)	0.726
Least disadvantaged	77.2 (74.7, 79.8)	0.356	68.9 (65.6, 72.2)	0.618	70.2 (66.4, 74.0)	0.762	67.5 (62.9, 72.1)	0.107	65.2 (59.6, 70.8)	0.096
Area of residence										
Major cities	76.6 (75.0, 78.3)		69.0 (66.9, 71.1)		68.4 (66.0, 70.9)		67.8 (64.9, 70.7)		68.0 (64.5, 71.5)	
Inner regional	78.2 (76.3, 80.2)	0.225	70.5 (67.9, 73.0)	0.384	73.0 (70.2, 75.9)	0.018	73.1 (69.7, 76.4)	0.023	72.0 (67.9, 76.1)	0.159
Outer regional/ remote/very remote	78.1 (74.8, 81.3)	0.452	73.4 (69.3, 77.6)	0.071	72.5 (67.6, 77.4)	0.156	72.2 (66.4, 78.0)	0.201	67.8 (60.6, 75.1)	0.960
**Glucagon-like peptide-1 receptor agonist**										
N	1347		700		402		244		131	
Sex										
Men	79.2 (76.0, 82.3)		62.2 (57.1, 67.3)		63.8 (57.2, 70.4)		63.9 (55.5, 72.2)		66.7 (55.5, 77.9)	
Women	83.1 (80.3, 85.9)	0.068	71.1 (66.4, 75.8)	0.013	66.6 (60.0, 73.1)	0.564	68.3 (59.9, 76.7)	0.471	66.1 (54.8, 77.4)	0.939
Age										
45-54 years	79.0 (72.1, 85.8)		74.0 (64.6, 83.3)		83.8 (72.7, 94.9)		70.9 (51.1, 90.7)		46.8 (14.5, 79.1)	
55-64 years	78.5 (75.3, 81.7)	0.898	63.6 (58.4, 68.7)	0.072	61.2 (54.2, 68.3)	0.007	61.9 (52.9, 70.9)	0.441	67.6 (56.4, 78.8)	0.331
65-74 years	85.3 (82.1, 88.6)	0.081	66.9 (60.9, 72.9)	0.237	63.3 (55.4, 71.2)	0.015	68.1 (58.9, 77.3)	0.806	68.2 (54.8, 81.6)	0.226
≥75 years	82.6 (75.3, 90.0)	0.484	75.6 (62.5, 88.7)	0.848	72.9, 55.6, 90.3)	0.290	77.3 (54.9, 99.8)	0.682	68.1 (39.0, 97.3)	0.353
Duration of diabetes										
<10 years	80.5 (77.1, 83.9)		62.4 (56.2, 68.6)		67.8 (59.8, 75.9)		60.3 (49.3, 71.4)		58.5 (42.3, 74.7)	
10-19 years	81.8 (78.8, 84.8)	0.569	69.5 (64.8, 74.3)	0.073	62.6 (56.2, 69.1)	0.331	67.3 (59.3, 75.2)	0.315	72.4 (62.4, 82.5)	0.154
≥20 years	81.1 (75.5, 86.6)	0.871	66.9 (57.8, 76.0)	0.430	68.6 (56.5, 80.6)	0.922	74.2 (58.8, 89.6)	0.178	56.9 (34.3, 79.5)	0.907

Socioeconomic status										
Most disadvantaged	81.8 (78.5, 85.1)		68.5 (63.1, 73.8)		67.5 (60.3, 74.7)		67.7 (58.4, 76.9)		71.5 (59.4, 83.7)	
Middle	80.6 (77.2, 84.0)	0.629	61.4 (55.6, 67.2)	0.083	66.0 (58.7, 73.4)	0.778	67.2 (57.8, 76.6)	0.947	63.8 (50.7, 77.0)	0.417
Least disadvantaged	81.3 (76.8, 85.8)	0.869	72.9 (65.5, 80.1)	0.367	58.9 (48.0, 69.8)	0.204	60.1 (46.0, 74.3)	0.386	60.9 (42.0, 79.7)	0.366
Area of residence										
Major cities	78.3 (75.1, 81.4)		64.1 (58.9, 69.3)		63.7 (57.0, 70.5)		64.7 (55.7, 73.7)		68.1 (56.4, 79.7)	
Inner regional	84.5 (81.3, 87.8)	0.010	69.4 (63.7, 75.0)	0.190	67.9 (60.4, 75.4)	0.428	67.6 (58.4, 76.7)	0.669	71.2 (58.6, 83.7)	0.730
Outer regional/ remote/very remote	83.5 (78.1, 88.8)	0.128	68.3 (59.9, 76.7)	0.421	63.2 (50.5, 75.9)	0.941	65.5 (49.0, 81.8)	0.945	47.4 (23.8, 71.1)	0.139
**Sodium-glucose cotransporter-2 inhibitor**										
N	5027		2917		1943		1023		475	
Sex										
Men	78.3 (76.7, 79.9)		67.8 (65.6, 69.9)		66.6 (63.9, 69.2)		70.3 (66.8, 73.9)		68.3 (63.0, 73.6)	
Women	79.1 (77.3, 81.0)	0.505	66.9 (64.1, 69.6)	0.620	66.9 (63.6, 70.3)	0.873	70.0 (65.4, 74.5)	0.901	68.0 (61.1, 74.9)	0.948
Age										
45-54 years	75.0 (70.7, 79.4)		62.2 (56.4, 68.1)		67.4 (60.5, 74.4)		65.9 (56.4, 75.5)		63.6 (48.7, 78.5)	
55-64 years	76.5 (74.6, 78.4)	0.538	64.9 (62.3, 67.5)	0.418	61.2 (58.0, 64.4)	0.121	68.6 (64.4, 72.7)	0.614	64.6 (58.2, 71.0)	0.906
65-74 years	80.9 (79.1, 82.8)	0.009	70.8 (68.1, 73.5)	0.007	71.9 (69.6, 75.2)	0.243	71.6 (67.0, 76.1)	0.287	71.6 (65.0, 78.3)	0.320
≥75 years	81.3 (77.9, 84.7)	0.029	71.3 (65.7, 76.9)	0.031	72.6 (65.5, 79.7)	0.312	78.5 (69.5, 87.6)	0.070	77.2 (64.0, 90.4)	0.194
Duration of diabetes										
<10 years	78.0 (76.1, 79.9)		66.6 (63.9, 69.4)		66.4 (63.0, 69.8)		68.5 (63.6, 73.3)		66.9 (59.7, 74.1)	
10-19 years	78.8 (77.0, 80.6)	0.552	67.5 (65.0, 70.0)	0.640	67.7 (64.7, 70.8)	0.571	72.6 (68.8, 76.4)	0.188	70.0 (64.2, 75.7)	0.511

≥20 years	79.9 (76.8, 83.0)	0.320	69.2 (64.9, 73.5)	0.329	64.3 (59.0, 69.7)	0.526	65.9 (58.3, 73.5)	0.574	65.0 (53.1, 76.8)	0.787
Socioeconomic status										
Most disadvantaged	78.7 (76.7, 80.7)		68.0 (65.2, 70.8)		66.1 (62.6, 69.6)		66.2 (61.4, 71.1)		68.3 (61.5, 75.1)	
Middle	78.8 (76.9, 80.7)	0.955	69.0 (66.3, 71.7)	0.598	68.2 (64.9, 71.5)	0.407	71.7 (67.3, 76.1)	0.101	69.2 (62.7, 75.8)	0.843
Least disadvantaged	78.2 (75.7, 80.7)	0.779	64.0 (60.4, 67.7)	0.100	65.2 (60.9, 69.6)	0.762	73.6 (68.1, 79.1)	0.059	66.2 (56.8, 75.5)	0.722
Area of residence										
Major cities	76.7 (75.0, 78.4)		65.7 (63.4, 68.0)		64.8 (61.9, 67.6)		66.3 (62.3, 70.2)		66.1 (60.1, 72.1)	
Inner regional	81.5 (79.5, 83.5)	0.001	71.0 (68.0, 73.9)	0.007	68.8 (65.1, 72.5)	0.098	74.1 (69.4, 78.8)	0.016	70.6 (63.8, 77.4)	0.343
Outer regional/ remote/very remote	79.6 (76.2, 83.1)	0.155	66.2 (61.2, 71.2)	0.857	70.4 (64.5, 76.3)	0.106	77.3 (69.8, 84.8)	0.021	70.2 (57.9, 82.5)	0.567

†Adjusted for sex, age in 2013, duration of diabetes in 2013, socioeconomic status at baseline and area of residence at baseline
